# Pro-Inflammatory Activation Promotes Atherogenic Endothelial Phenotype in Male and Female Human Umbilical Endothelial Vein Cells (HUVECs)

**DOI:** 10.3390/ijms27073079

**Published:** 2026-03-27

**Authors:** Mario Lorenz, Riwka Palant, Edith Oscherowa, Weam Karmid-Haj Hamoud, Jennifer A. Kirwan, Sarah Trajkovski, Janine Wiebach, Raphaela Fritsche-Guenther, Haiyan Wu, Natalie Haritonow, Angelika Vietzke, Elena Kaschina, Wolfgang Henrich, Julia Temp, Maria Luisa Barcena

**Affiliations:** 1 Charité–Universitätsmedizin Berlin, Corporate Member of Freie Universität Berlin and Humboldt-Universität zu Berlin, Charitéplatz 1, 10117 Berlin, Germany; 2 Deutsches Herzzentrum der Charité, Klinik für Kardiologie, Angiologie und Intensivmedizin, CCM, Charitéplatz 1, 10117 Berlin, Germany; 3 DZHK (German Centre for Cardiovascular Research), Partner Site Berlin, 10785 Berlin, Germany; 4 Department of Urology, Eberhard Karls University of Tuebingen, 72076 Tuebingen, Germany; 5 Department of Geriatrics and Medical Gerontology, Charité–Universitätsmedizin Berlin, Corporate Member of Freie Universität Berlin and Humboldt-Universität zu Berlin, Charitéplatz 1, 10117 Berlin, Germany; 6 Berlin Institute of Health at Charité–Universitätsmedizin Berlin, BIH Metabolomics, Charitéplatz 1, 10117 Berlin, Germany; 7 Max Delbrück Center for Molecular Medicine in the Helmholtz Association, Robert-Rössle-Str. 10, 13125 Berlin, Germany; 8 University of Veterinary Medicine Vienna, Veterinaerplatz 1, 1210 Vienna, Austria; 9 Department of Respiratory, Zhejiang Hospital, Hangzhou 310058, China; 10 Institute of Pharmacology, Max Rubner Center for Cardiovascular Metabolic Renal Research (MRC), 10115 Berlin, Germany; 11 Department of Obstetrics, Charité–Universitätsmedizin Berlin, Corporate Member of Freie Universität Berlin and Humboldt-Universität zu Berlin, Charitéplatz 1, 10117 Berlin, Germany; 12 Faculty of Medicine, Danube Private University, 3500 Krems an der Donau, Austria

**Keywords:** sex differences, human umbilical vein endothelial cells (HUVECs), inflammation, cytokines, focal proteins

## Abstract

Atherosclerosis, the leading global cause of death, is a chronic inflammatory vascular disease with higher prevalence and earlier onset in men than in women. This study aims to investigate sex differences in the atherogenic endothelial phenotype during early atherosclerosis processes by providing the first comprehensive analysis of hormone-independent responses in human umbilical vein endothelial cells (HUVECs) from opposite-sex twins. HUVECs underwent pro-inflammatory stimulation with TNF-α and supernatant from activated pro-inflammatory THP-1 cells, revealing distinct sex-specific patterns: mRNA expression of focal adhesion proteins *talin-I*, *vinculin*, *FAK*, and *α1-actinin* increased significantly only in male cells, while paxillin showed elevated mRNA and protein levels in both sexes. Male HUVECs exhibited stronger induction of cell adhesion molecule *VCAM-1*, pro-inflammatory cytokine IL-1β, and proangiogenic factors Flt-3L, G-CSF, and PDGF-AA, whereas IL-22 secretion was exclusively upregulated in female cells. These sex differences in levels of focal adhesion, adhesion molecules, and cytokine profiles uncover the mechanistic backgrounds of the atherogenic endothelial phenotype, independent of systemic hormones. The findings emphasize cellular sex as a critical biological variable in early atherosclerosis and vascular inflammation.

## 1. Introduction

Cardiovascular diseases (CVD) are the leading global cause of death, claiming approximately 19.2 million lives in 2023 [1]. Men exhibit higher mortality rates than women, particularly in Eastern and Central Europe and in Central Asia [2]. Disease manifestations differ markedly between sexes: women more frequently experience cerebrovascular disease or heart failure as the initial event, whereas men predominantly develop coronary heart disease, with myocardial infarctions prevailing in men until the seventh decade [3,4]. Although men show higher prevalence of traditional risk factors such as smoking, hypertension, and type 2 diabetes [5], these factors confer a disproportionately greater cardiovascular risk in women, particularly diabetic women with elevated rates of CVD mortality, myocardial infarction and ischemic stroke [6]. Atherosclerosis is classified as a chronic inflammatory and fibroproliferative disease characterized by focal intimal lipid and calcium deposits in medium and large arteries [7]. Current therapies, including statins and PCSK9 inhibitors for cholesterol reduction alongside antihypertensives, mitigate risk factors and slow disease progression [8,9]. However, so far, they have failed to address core pathophysiological mechanisms. Endothelial injury triggered by oxidized LDL (oxLDL), hyperglycemia, disturbed shear stress at arterial branches, and hypertension increases intimal permeability and stimulates the release of atherogenic cytokines, chemokines, and growth factors [10,11,12,13,14]. These mediators recruit monocytes via the adhesion molecules ICAM-1 and VCAM-1 to the endothelium [15]. Monocytes then differentiate after transmigration into pro-inflammatory M1 macrophages that form foam cells through cholesterol uptake, while apoptosis and necrosis contribute to lipid core formation [16]. T helper (Th)1 cytokines such as TNF-α and IL-6 drive atherogenesis [17,18], whereas anti-inflammatory mediators like IL-10, IL-1RA, and TGF-β exert protective effects [19,20,21]. An imbalance of growth factors (PDGF/TGF-β/M-CSF) destabilizes plaques through matrix metalloproteinases (MMPs), matrix degradation, and neovascularization [22,23]. Elevated lipoprotein(a) further promotes pro-inflammatory monocyte transmigration and cytokine secretion [24]. This is seen in LPS-stimulated monocytes from coronary atherosclerosis patients [25].

Sex differences in atherosclerosis arise from multiple causes. Hormonal factors, particularly the protective effects of estrogens, and genetic regulation contribute to these differences [26,27]. For example, studies in human umbilical vein endothelial cells (HUVECs) from dizygotic twins reveal innate sex-specific differences in mRNA and protein expression under conditions of serum deprivation or mechanical stress [28]. Focal adhesion proteins regulate endothelial barrier function, migration, and permeability [29,30], and their dysfunction promotes atherosclerosis [31]. Sex differences in focal adhesion proteins could therefore influence the progression of atherosclerosis. Different studies revealed a sex-specific regulation. Female HUVECs upregulate phosphorylated focal adhesion kinase (FAK) under estrogen stimulation [32], while the vinculin SNP rs4746172 is associated with male hypertension [33]. The adhesion molecules VCAM-1 and ICAM-1 mediate leukocyte transmigration via TNF-α/IL-1β/LDL/ROS pathways [34,35,36,37], with VCAM-1 inhibition showing promise as a therapeutic target for plaque reduction [38]. Sex differences were also described; lower VCAM-1 expression promoted female survival in shock models [39]. For secreted cytokines, male mice with myocarditis typically produce pro-inflammatory cytokines (IL-1β and IL-18), whereas females secrete anti-inflammatory IL-4 [40,41]. Clinical data show elevated IL-6 and MCP-1 in women with CHD [42].

Against this background, we hypothesized that male and female endothelial cells exhibit distinct, hormone-independent responses to pro-inflammatory stimulation that differentially shape an atherogenic endothelial phenotype. Specifically, we expected that the exposure of twin-derived male and female HUVECs to TNF-α or conditioned medium from activated THP-1 cells would reveal sex-dependent differences in the expression of focal adhesion proteins and adhesion molecules, as well as in cytokine and growth factor secretion profiles. By systematically comparing these responses between male and female cells, our aim was to identify early, cell-intrinsic sex differences in endothelial activation that could contribute to the divergent course of atherosclerosis in men and women.

## 2. Results

### 2.1. Sex Differences in Focal Adhesion Protein Expression in Activated HUVECs

**Figure 1:**
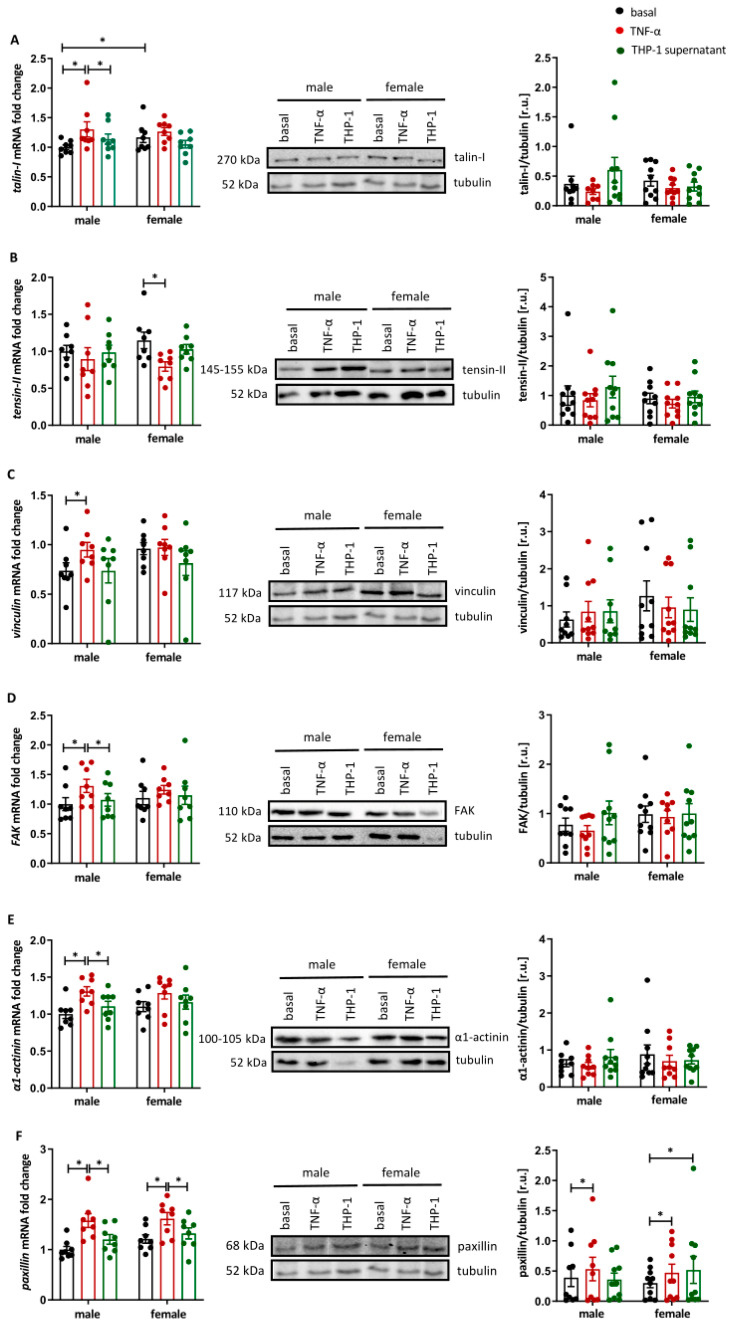
Sex differences in the expression of focal adhesion proteins. Real-time PCR, Western blots and corresponding densitometric analysis of (**A**) talin-I, (**B**) tensin-II, (**C**) vinculin, (**D**) FAK, (**E**) α1-actinin, and (**F**) paxillin from male and female HUVECs treated with TNF-α (10 ng/mL) or supernatant from pro-inflammatory THP-1 cells (1:1 dilution in cell culture medium) for 24 h. Data are presented as bar charts with individual data dots and the means ± SEM (PCR: n = 8 and Western blot n = 10). All data were normalized to the mean of the male basal control, and Western blot data are expressed in relative units (r.u.). For all individual Western blots, samples were run on the same gel. Statistical analysis was performed using the paired Wilcoxon test. * *p* < 0.05. basal: control; TNF-α: TNF-α treatment; THP-1 supernatant: THP-1 supernatant treatment.

Successful M1 polarization of THP-1 cells was confirmed by increased IL-6, MIP-1β, and TNF-α secretion. Focal adhesion proteins play a pivotal role in the development of atherosclerosis [31]. To investigate sex-specific differences in the expression of prominent focal adhesion proteins in activated endothelial cells, male and female HUVECs were treated with TNF-α or the supernatant of pro-inflammatory THP-1 cells. TNF-α significantly increased the *talin-I* expression at the mRNA level in male HUVECs (Figure 1A). In addition, *tensin-II* mRNA expression was significantly reduced in female HUVECs after TNF-α treatment (*p* < 0.05) (Figure 1B). In contrast, the mRNA expression of *vinculin*, *FAK*, *α-actinin* and *paxillin* was significantly increased in male HUVECs following TNF-α stimulation (*p* < 0.05), while in female HUVECs, the pro-inflammatory stimulus induced only *paxillin* mRNA expression (*p* < 0.05) (Figure 1C–F). Treatment with the supernatant from pro-inflammatory THP-1 cells did not alter the mRNA expression of any focal adhesion protein compared to the control in either male or female HUVECs (Figure 1A–F). At the protein level, TNF-α increased paxillin expression in both male and female HUVECs (*p* < 0.05), whereas protein levels of talin-I, tensin-II, vinculin, FAK and α-actinin were not affected (Figure 1A–F).

### 2.2. Pro-Inflammatory Stimulus Promotes the Expression of Adhesion Molecules and Mitochondrial ROS Formation in Male and Female HUVECs

**Figure 2:**
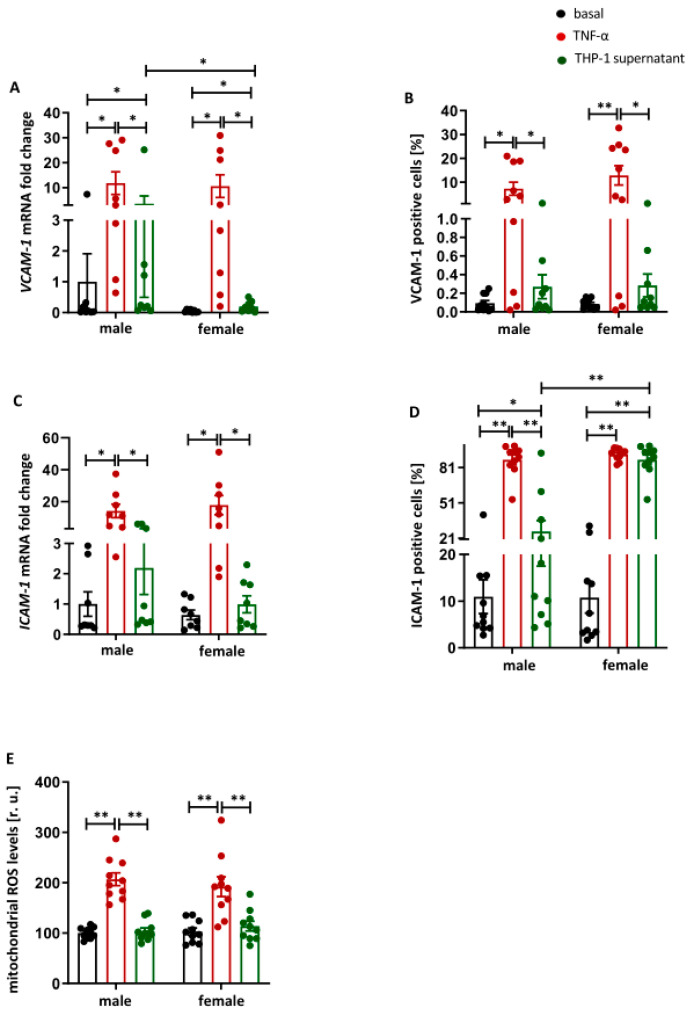
Pro-inflammatory stimuli promote the expression of adhesion molecules and mitochondrial ROS formation in male and female HUVECs. Male and female HUVECs were treated with TNF-α (10 ng/mL) or supernatant from pro-inflammatory THP-1 cells (1:1 dilution in cell culture medium) for 24 h. Real-time PCR analyses and flow cytometry (**A**,**B**) of VCAM-1 and (**C**,**D**) ICAM-1 and (**E**) mitochondrial ROS formation (MitoSOX assay). Data are presented as bar charts with individual data dots and means ± SEM (PCR: n = 8; FACS and MitoSOX n = 10). All data were normalized to the mean of the male basal control. Statistical analysis was performed using the paired Wilcoxon test. * *p* < 0.05, ** *p* < 0.01. basal: control; TNF-α: TNF-α treatment; THP-1 supernatant: THP-1 supernatant treatment.

Since focal adhesion proteins modulated the expression of endothelial adhesion molecules VCAM-1 and ICAM-1, we next assessed potential sex-specific effects of pro-inflammatory stimulation (TNF-α or supernatant from pro-inflammatory THP-1 cells) in male and female HUVECs. Both TNF-α and THP-1 supernatant significantly increased *VCAM-1* mRNA expression in male and female HUVECs (*p* < 0.05) (Figure 2A), whereas VCAM-1 protein levels were only elevated following TNF-α treatment in both sexes (*p* < 0.05 and *p* < 0.01) (Figure 2B and Supplementary Materials Figure S1B,C). ICAM-1 mRNA and protein expression were significantly upregulated by TNF-α in both male and female HUVECs (*p* < 0.05 and *p* < 0.01) (Figure 2C,D and Figure S1B,C). At the protein level, treatment with THP-1 supernatant also significantly increased ICAM-1 expression in HUVECs of both sexes (*p* < 0.05 and *p* < 0.01) (Figure 2D and Figure S1B,D), although the effect was more pronounced in female cells (*p* < 0.05) (Figure 2D and Figure S1B,D). We also measured mitochondrial ROS levels. TNF-α treatment significantly increased mitochondrial ROS formation in both male and female HUVECs (*p* < 0.01) (Figure 2E).

### 2.3. Pro-Inflammatory Stimulus Promotes the Secretion of Atherosclerosis-Related Cytokines and Chemokines in Male and Female HUVECs

**Figure 3:**
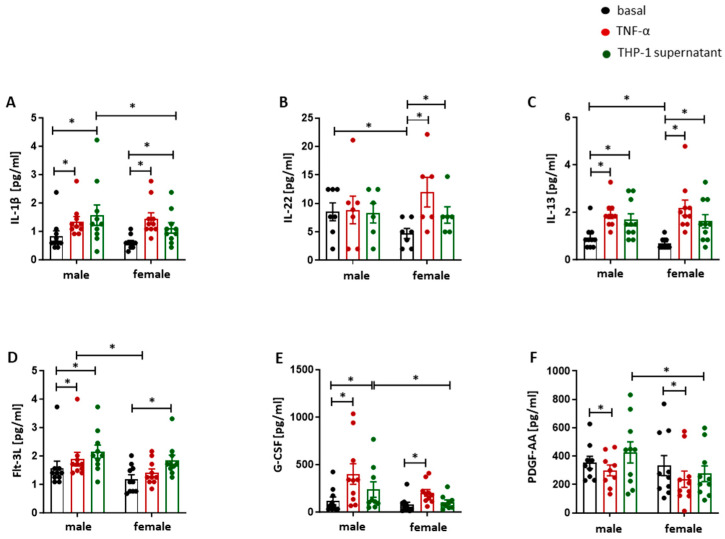
Induced secretion of atherogenic cytokines and growth factors in activated male and female HUVECs. MAGPIX^®^ analyses of (**A**) IL-1β, (**B**) IL-22, (**C**) IL-13, (**D**) Flt-3L, (**E**) G-CSF, and (**F**) PDGF-AA in supernatants from male and female HUVECs treated with TNF-α (10 ng/mL) or supernatant from pro-inflammatory THP-1 cells (1:1 dilution in cell culture medium) for 24 h. Data are presented as bar charts with individual data dots and means ± SEM (n = 10). All data were normalized to the mean of the male basal control. Statistical analysis was performed using the paired Wilcoxon test. * *p* < 0.05. basal: control; TNF-α: TNF-α treatment; THP-1 supernatant: THP-1 supernatant treatment.

**Figure 4:**
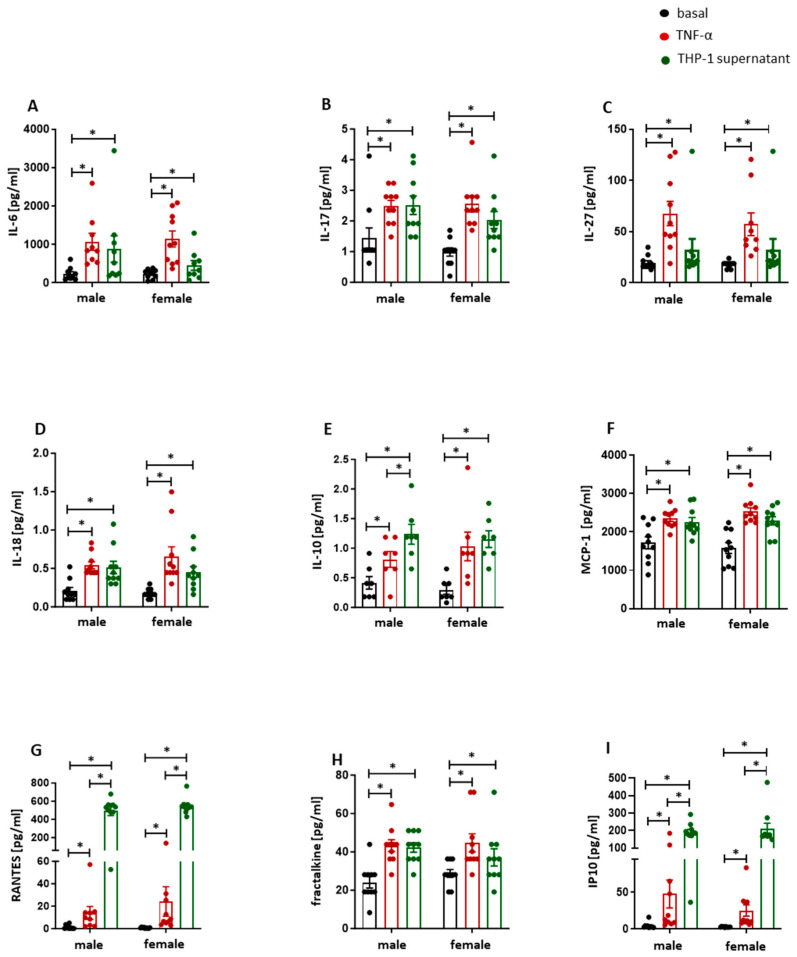
Induced secretion of atherogenic cytokines and chemokines in activated male and female HUVECs. MAGPIX^®^ analyses of (**A**) IL-6, (**B**) IL-17, (**C**) IL-27, (**D**) IL-18, (**E**) IL-10, (**F**) MCP-1, (**G**) RANTES, (**H**) fractalkine, and (**I**) IP-10 in supernatants from male and female HUVECs treated with TNF-α (10 ng/mL) or supernatant from pro-inflammatory THP-1 cells (1:1 dilution in cell culture medium) for 24 h. Data are presented as bar charts with individual data dots and means ± SEM (n = 10). All data were normalized to the mean of the male basal control. Statistical analysis was performed using the paired Wilcoxon test. * *p* < 0.05. basal: control; TNF-α: TNF-α treatment; THP-1 supernatant: THP-1 supernatant treatment.

Since pro-inflammatory activation stimulated an adhesive phenotype and ROS formation in male and female HUVECs, we then determined the secretion of key atherosclerosis-related mediators in the cell culture supernatants of male and female endothelial cells. The most prominent sex-dependent effects were observed for IL-1β, IL-22, IL-13 and selected growth factors. Male HUVECs secreted significantly higher levels of the pro-inflammatory cytokine IL-1β and the proangiogenic growth factors Flt-3L, G-CSF and PDGF-AA after TNF-α or THP-1 stimulation, whereas IL-22 secretion was selectively increased in activated female HUVECs under both conditions. In addition, at baseline, the anti-inflammatory cytokine IL-13 was more abundant in supernatants from male HUVECs (Figure 3A–F). Beyond these mediators with clear sex differences, TNF-α and THP-1 supernatant also induced a broad, largely sex-independent pro-inflammatory profile. HUVECs treated with either TNF-α or supernatant from pro-inflammatory THP-1 cells secreted a panel of atherogenic cytokines (IL-6, IL-17, IL-27, IL-18 and IL-10) and chemokines (MCP-1, RANTES, fractalkine and IP-10) in a sex-independent manner (Figure 4A–I). Other pro-inflammatory cytokines such as IL-1α, IL-3, IL-5, IL-12(p70), IL-17F, sCD40L, and TNF-β were elevated in cell culture medium from male and female HUVECs after TNF-α or treatment with supernatant from pro-inflammatory THP-1 cells, without sex differences (Figure S2A–G). The levels of the anti-inflammatory cytokines IL-1RA and IL-4 were also significantly increased in male and female HUVECs after treatment with TNF-α or supernatant from pro-inflammatory THP-1 (*p* < 0.05), where IL-1RA levels were higher after stimulation with THP-1 supernatant compared with TNF-α in both sexes (*p* < 0.05) (Figure S2H,I). A similar pattern was observed for cytokines with pro- as well as anti-inflammatory properties, like INF-α2, IL-2, IL-12(p40), and IL-17E (*p* < 0.05) (Figure S2J–M).

The secretion of various chemokines such as eotaxin, GROα, IL-8, MIG, and MIP-1β was also markedly modulated in response to TNF-α and THP-1 supernatant (Figure S3A–E). This was likewise observed for different growth factors, including FGF-2, IL-9, IL-15, M-CSF, PDGF-AB/BB and TGF-α (*p* < 0.05) (Figure S3F–K).

## 3. Discussion

The primary aim of this study was to investigate potential sex-specific differences in the atherogenic endothelial phenotype in activated HUVECs. The main findings are as follows: (i) In both male and female HUVECs, a pro-inflammatory environment stimulates the expression of focal adhesion proteins, supporting endothelial activation in atherosclerosis models. (ii) Adhesion molecules such as VCAM-1 and ICAM-1 are upregulated, accompanied by increased mitochondrial ROS formation in activated male and female HUVECs, consistent with endothelial dysfunction in atherogenesis. (iii) Atherogenic cytokines and chemokines are secreted more abundantly by activated HUVECs under pro-inflammatory conditions, indicating a shift toward a pro-inflammatory, pro-atherogenic secretory profile. Observed sex differences could alter the incidence and development of atherosclerosis.

Atherosclerosis, the leading cause of death worldwide, is a chronic inflammatory disease of the vascular system, with men showing a higher prevalence and earlier clinical manifestation than women [3]. While most of the earlier work has largely focused on hormonal factors, cellular and molecular sex differences in the vasculature have been comparatively understudied, despite endothelial dysfunction and monocyte adhesion representing early key events in atherogenesis [43]. To address these gaps, this study established a human in vitro model of early atherogenesis using twin-derived HUVECs stimulated with TNF-α or conditioned medium from activated THP-1 monocytes, a well-established model for peripheral blood mononuclear cells [44]. The use of genetically related donors can reduce inter-individual variability. HUVECs are a widely used cell model in cardiovascular research and they recapitulate key aspects of human endothelial biology [45]. Under our experimental conditions, sex-specific differences in focal adhesion proteins, adhesion molecules and cytokine secretion were identified at early atherosclerotic conditions, suggesting differential endothelial signaling in male and female cells at molecular levels. The findings of this study indicate a layered, sex-specific regulation of endothelial activation under pro-inflammatory, early atherogenic conditions. Specifically, the focal adhesion proteins talin-1, vinculin, FAK and α1-actinin were significantly upregulated at the mRNA level exclusively in male endothelial cells following pro-inflammatory stimulation, whereas paxillin was induced at both the transcript and protein level in male and female HUVECs. Focal adhesions physically link the extracellular matrix to the actin cytoskeleton and orchestrate adhesion, migration, survival and gene expression in endothelial cells [29,30]. The stronger upregulation of talin-1, vinculin, FAK and α1-actinin transcripts in male HUVECs under atherogenic stimulation is consistent with emerging data linking focal adhesion components and integrin signaling to sex-dependent vascular phenotypes and cardiovascular risk [32].

Talin-1, tensin-II, vinculin, FAK, and α1-actinin remained unchanged at the protein level, whereas paxillin increased in both sexes. The discrepancy between mRNA and total protein levels observed for most focal adhesion proteins underscores the relevance of post-transcriptional and post-translational regulation and modifications [46,47]. In this line, previous work has shown that TNF-α modulates the phosphorylation and redistribution of FAK and paxillin rather than their bulk abundance [48,49]. In our model, pro-inflammatory stimulation induced sex-dependent changes in the transcript levels of several focal adhesion-related genes, whereas these effects were only partially mirrored at the level of total protein abundance, with paxillin being the only focal adhesion protein showing a consistent increase in both mRNA and protein levels. This apparent discrepancy between mRNA and protein levels is consistent with the notion that focal adhesion proteins are strongly regulated by post-transcriptional and post-translational mechanisms, including phosphorylation, subcellular redistribution and dynamic complex assembly, rather than by changes in bulk protein content [50]. Our findings therefore suggest that sex-dependent regulation of the endothelial adhesion machinery under early atherogenic conditions may manifest more prominently at the level of protein activation and focal adhesion remodeling than at the level of overall protein expression.

Since focal adhesion proteins drive the upregulation of the adhesion molecules ICAM-1 and VCAM-1 [51,52], the present study next examined whether these endothelial adhesion proteins exhibit sex-specific regulation. ICAM-1 and VCAM-1 are crucial for leukocyte adhesion to the endothelium, an early step in the formation of inflammatory vascular lesions and a key contributor to atherosclerosis progression [53,54]. In this model, TNF-α and conditioned medium from activated THP-1 cells strongly increased ICAM-1 and VCAM-1 mRNA and protein levels in male and female HUVECs, confirming their central role in pro-inflammatory endothelial activation. These findings support their relevance as therapeutic targets whose inhibition can attenuate vascular inflammation. Consistent with these findings, previous studies have shown that various pro-inflammatory cytokines and LPS induce ICAM-1 and VCAM-1 expression in endothelial cells [55]. A key finding of our study is the sex-specific regulation of VCAM-1. After THP-1 supernatant stimulation, VCAM-1 expression was significantly higher in male compared to female endothelial cells, whereas ICAM-1 showed no sex differences. This is consistent with clinical data reporting elevated VCAM-1, but not ICAM-1, in male adolescents with metabolic risk or subclinical atherosclerosis [56]. In the context of the increase in focal adhesion molecules preferentially in males, these results support the concept that VCAM-1, rather than ICAM-1, could be a critical endothelial effector linking male-specific focal adhesion signaling to enhanced leukocyte recruitment and a greater atherogenic burden.

**Figure 5:**
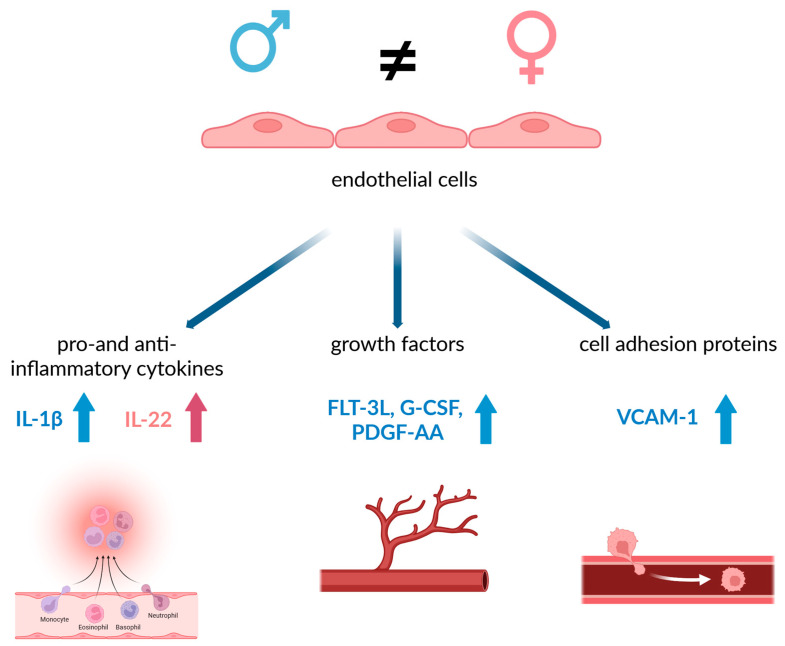
Endothelial cells differ in a sex-specific manner in the secretion of the pro- and anti-inflammatory cytokines IL-1β and IL-22; in the secretion of the growth factors Flt-3L, G-CSF and PDGF-AA; and in the expression of the cell adhesion protein VCAM-1. These sex differences at the cellular level may have an impact on early stages of atherogenic processes.

The multiplex analysis revealed largely sex-independent regulation of most cytokines and chemokines in response to TNF-α and THP-1-derived mediators, indicating a shared inflammatory response. However, key sex-specific differences emerged. Secretion of IL-1β, a prototypical pro-atherogenic cytokine, was higher in male HUVECs, consistent with experimental data implicating IL-1 signaling in plaque formation and progression [57]. Among growth factors, Flt-3L, G-CSF and PDGF-AA were more strongly induced in male cells, suggesting a male-biased amplification of endothelial–immune crosstalk, myeloid cell activation and vascular remodeling pathways that are known to promote plaque growth and instability. In contrast, IL-22 secretion was selectively increased in activated female HUVECs. IL-22 has context-dependent roles in vascular biology, with evidence for both pro-inflammatory, plaque-promoting actions and barrier-protective effects on endothelial cells [58]. The preferential IL-22 induction in female cells may therefore reflect a distinct, potentially dual endothelial response in early atherogenesis that warrants further mechanistic exploration. Figure 5 summarizes the observed sex differences in secreted factors and adhesion molecules.

### Limitations

This study has several limitations. First, the sample size of opposite-sex twin pairs was relatively small, which may limit statistical power and the generalizability of our findings, although the paired design within twin donors partially mitigates inter-individual variability. In addition, outlier exclusion was performed on a small dataset, and thus, although it did not change the overall conclusions, the results should be interpreted with this aspect in mind. Second, our analyses focused on markers of endothelial activation, including focal adhesions, adhesion molecules, and cytokine and growth factor secretion, as well as mitochondrial ROS. We did not integrate functional endothelial assays such as leukocyte adhesion, permeability or migration. Thus, while the observed sex-dependent patterns are compatible with differences in atherogenic endothelial behavior, we cannot directly demonstrate cellular parameters like barrier function or leukocyte–endothelium interaction. Future studies should incorporate targeted functional experiments to confirm the physiological relevance of these findings.

Third, all experiments were conducted under static culture conditions, even though hemodynamic shear stress is a key regulator of endothelial signaling, barrier properties and inflammatory activation and strongly modulates atherogenesis. Our results therefore need to be validated in flow-based models with physiological and disturbed shear stress. Finally, we used venous endothelial cells from umbilical cords. Accumulating single-cell and bulk-omics data have demonstrated pronounced endothelial heterogeneity across vascular beds, including relevant differences between venous and arterial endothelial cells. Therefore, the sex-specific signatures identified here should be confirmed in arterial endothelial models such as human coronary or aortic endothelial cells to establish their relevance for adult atherosclerosis-prone vessels.

## 4. Materials and Methods

### 4.1. Isolation and Cultivation of HUVECs

Male and female HUVECs from dizygotic twins (n = 10 pairs) were isolated from umbilical cord veins by digestion at 37 °C for 20 min with collagenase type II (0.5 mg/mL, Biochrom KG, Berlin, Germany). Flushing the veins twice with Hank’s buffered salt solution (HBSS) released the detached cells. They were then centrifuged for 5 min at 1200× *g* and cell pellets were resuspended in medium M199 (Thermo Fisher Scientific, Waltham, MA, USA). Cells were cultivated in M199 medium supplemented with 20% fetal calf serum (Sigma-Aldrich, St. Louis, MO, USA), 12 μg/mL endothelial cell growth supplement (ECGS; PromoCell, Heidelberg, Germany), 5 U/mL heparin (HIMEDIA, Thane, Maharashtra, India), 2 mM L-glutamine (Thermo Fisher Scientific, Waltham, MA, USA), 5 μg/mL ascorbic acid (Sigma-Aldrich, St. Louis, MO, USA), 5 μg/mL glutathione (Sigma-Aldrich, St. Louis, MO, USA), 100 U/mL penicillin (Thermo Fisher Scientific, Waltham, MA, USA), 100 μg/mL streptomycin (Thermo Fisher Scientific, Waltham, MA, USA), and 50 ng/mL amphotericin B (Biochrom, Merck, Berlin, Germany). Cultures were incubated at 37 °C in a humidified atmosphere with 5% CO_2_. For sex determination, mRNA levels of SRY (sex-determining region Y that is expressed from the Y chromosome only) and XIST (X-inactive-specific transcript, expression is restricted to female cells) were determined by TaqMan Gene Expression Assays from Applied Biosystems (SRY Hs00976796, XIST Hs0179824, Waltham, MA, USA), and beta-actin (Hs01060665; Thermo Fisher Scientific, Waltham, MA, USA) served as the reference gene. Isolation of male and female HUVECs from dizygotic twin pairs of the opposite sex was approved by the Charité University Hospital Ethics Committee (EA2/017/13) and conformed to local university guidelines and those outlined in the Declaration of Helsinki. The mothers provided their written informed consent.

### 4.2. Pro-Inflammatory Activation of Monocytic-like Cells

Human THP-1 cells (ATCC^®^ TIB-202™, Manassas, VA, USA) were cultivated in RPMI 1640 medium (Gibco, Gaithersburg, Germany) with L-glutamine, 10% Fetal Bovine Serum Advanced (Capricorn Scientific, Ebsdorfergrund, Germany), and 1% penicillin/streptomycin (Biochrom, Germany) at 37 °C and 5% CO_2_ [59]. Cells were harvested by centrifugation (5 min/300 rpm) and resuspended in fresh medium every 2 days. THP-1 cells were polarized into M1 monocyte-like cells with 10 ng/mL LPS (Sigma-Aldrich, Schnelldorf, Germany) and 10 ng/mL IFN-γ (PeproTech, Hamburg, Germany) for 24 h. The cell culture medium was collected and centrifuged at 1200 rpm for 5 min at 4 °C. The supernatants were stored at −80 °C. All experiments were performed in duplicate, and the experiments were repeated a minimum of five times to ensure replicability.

### 4.3. Treatment of HUVECs

In total, 5 × 10^5^ cells were seeded into 6-well plates. After reaching confluence, male and female HUVECs were activated with 10 ng/mL TNF-α (PeproTech, Hamburg, Germany) for 24 h. In parallel, HUVECs were cultivated with a 1:1 mixture of HUVEC medium and conditioned medium from M1-THP-1 cells for 24 h.

### 4.4. RNA Isolation and Quantitative Real-Time (RT)-PCR

Total RNA was isolated from HUVECs using TRIzol™ Reagent (Invitrogen, Carlsbad, CA, USA). Quantitative real-time PCR was performed with Power SYBR™ Green PCR Master Mix (Applied Biosystems, Waltham, MA, USA) for *VCAM-1* and *ICAM-1* and TaqMan (Applied Biosystems, Waltham, MA, USA) for *α1-actinin*, *FAK*, *paxillin*, *talin-I*, *tensin-II* and *vinculin* (Supplementary Materials Table S1). Relative target mRNA levels were calculated using the comparative threshold (Ct) method, normalized to the mean expression of *hypoxanthine phosphoribosyl transferase* (*HPRT*) and *ribosomal protein lateral stalk subunit P0* (*RPLP0*) (Supplementary Materials Table S1). Results are presented as fold change relative to the mean of the untreated male group after normalization.

### 4.5. Protein Extraction and Immunoblotting

Male and female HUVECs were homogenized in Laemmli buffer, as described [60]. Protein concentrations were determined using the BCA assay (Thermo Scientific Pierce Protein Biology, Dreieich, Germany). Equal amounts of total protein (15 µg) were separated on SDS-polyacrylamide gels and transferred to nitrocellulose membranes. Membranes were incubated overnight with the following primary antibodies: α1-actinin (1:5000, #66895-1-Ig, Proteintech, Rosemont, IL, USA), FAK (1:500, #126361-AP, Proteintech, Rosemont, IL, USA), paxillin (1:1000, #10029-1, Proteintech, Rosemont, IL, USA), talin-I (1:1000, #4021P, Cell Signaling, Danvers, MA, USA), tensin-II (1:1000, #11990S, Cell Signaling, Danvers, MA, USA), and vinculin (1:5000, #66305-1-Ig, Proteintech, Rosemont, IL, USA). Equal sample loading was verified using α-tubulin (1:1000, #3873, Cell Signaling, Danvers, MA, USA) or GAPDH (1:1000, #97166, Cell Signaling, Danvers, MA, USA). Immunoreactive bands were visualized with ECL Plus (GE Healthcare, Cardiff, UK) and quantified using ImageLab (Version 6.1, Bio-Rad Laboratories, Hercules, CA, USA). All male and female samples were run on the same gel.

### 4.6. ELISA for Determination of Single Cytokines

The supernatants of untreated and pro-inflammatory THP-1 cells were collected and 100 µL was used in ELISA assays for IL-6 (R&D Systems, Minneapolis, MN, USA), 200 µL for MIP-1β (R&D Systems, Minneapolis, MN, USA) and 100 µL for TNF-α (Proteintech, Rosemont, IL, USA) according to the manufacturer’s instructions.

### 4.7. MAGPIX^®^ Analysis

Cytokine levels in basal and stimulated HUVEC supernatants were measured using the MagPix multiplex analysis system (Merck, Darmstadt, Germany). Cell supernatants were frozen in liquid nitrogen after flow experiments and processed according to the manufacturer’s instructions for multiplex bead-based immunoassays. This method allows simultaneous quantification of multiple cytokines by capturing target proteins on fluorescently labeled beads, followed by detection using a Luminex xMAP technology-based instrument (Merck, Darmstadt, Germany), providing sensitive and specific measurement of secreted cytokines. The HCYTA-60K-PX48 Cytokine/Chemokine/Growth Factor Panel A Kit (Merck, Darmstadt, Germany) was used according to the manufacturer’s recommendations. For acquiring data, the xPONENT 4.2 software (Merck, Darmstadt, Germany) was used. All samples yielded a bead count >30 per analyte. Background signals from blank supernatants were subtracted from the mean fluorescent intensities (MFI) and analyte concentrations (pg/mL) were calculated from the linear range of a 7-point standard curve including matrix using Rpackage MSlineaR 0.2.2 [61]. To assess instrumental variance, pooled quality control (QC) samples were analyzed in triplicate at the beginning, middle and end of the run. The biological variability between samples exceeded the technical variability of the assay. Compounds with no linear range or more than 20% of missing values were excluded, which applies to EGF, GM-CSF, MCP-3, MIP-1alpha, VEGF-A, IL-7 and MDC. Additionally, TNF-α and IFN-γ were excluded, as they were used for the stimulation of HUVECs and THP-1, respectively. Of 48 cytokines included in the kit, 39 were used for data analysis.

### 4.8. Flow Cytometry

Adhesion molecule expression on activated HUVECs was analyzed by flow cytometry using the following antibodies: human anti-CD106-PE-Cyanine7 (VCAM-1) (Clone: STA, Cat# 25-1069-42, Thermo Fisher Scientific, Dreieich, Germany) and human anti-CD54-Alexa-Fluor770 (ICAM-1) (Clone: HA58, Cat# 353126, BioLegend, Koblenz, Germany), as previously described [62] (Figure S1A). Flow cytometry was performed using a MACSQuant Analyzer 10 (Miltenyi Biotec, Bergisch Gladbach, Germany), and data analysis was conducted with FlowJo software (Version 10.9).

### 4.9. Mitochondrial ROS Measurement

Mitochondrial reactive oxygen species (ROS) levels in male and female HUVECs were assessed 24 h after treatment with TNF-α or supernatant from pro-inflammatory THP-1 cells, following the manufacturer’s protocol. Cells were incubated with 5 µM MitoSox red for 30 min, washed twice with PBS containing 1 mM calcium chloride and lysed with a 0.5% TritonX-100 buffer. Fluorescence intensity was measured using a ViktorX Multilabel Plate reader (Perkin Elmer, Shelton, CT, USA) (excitation at 485 ± 10 nm and emission at 530 ± 10 nm) and normalized to protein content [60].

### 4.10. Statistical Analysis

Data are presented as fold changes with medians and interquartile ranges. For all analyses comparing male and female HUVECs, cells derived from the same opposite-sex twin pair were treated as matched biological samples, and comparisons were performed within each twin pair rather than between independent groups. Accordingly, statistical evaluation was carried out using the paired Wilcoxon test. Outliers were identified and removed from data analysis using the interquartile range (IQR) method, where values exceeding 1.5 × IQR below Q1 or above Q3 were excluded. Statistical analyses were performed using GraphPad Prism 10 (GraphPad Software, San Diego, USA). Statistical significance was accepted when *p* < 0.05.

## 5. Conclusions

This study reveals sex-specific differences in endothelial responses to inflammatory stimulation. Male cells exhibited stronger upregulation of several focal adhesion proteins and cytokines, whereas IL-22 secretion was higher in female cells. These findings establish endothelial sex as a critical biological variable in early atherosclerosis, warranting sex-specific therapeutic strategies.

## Data Availability

The original contributions presented in this study are included in the article/Supplementary Materials. Further inquiries can be directed to the corresponding author.

## Supplementary Materials

The following supporting information can be downloaded at: https://www.mdpi.com/article/10.3390/ijms27073079/s1.
