# Supplementary material for: Pro-Inflammatory Activation Promotes Atherogenic Endothelial Phenotype in Male and Female Human Umbilical Endothelial Vein Cells (HUVECs)
Source: Int J Mol Sci. 2026 Mar 27;27(7):3079. doi: 10.3390/ijms27073079 (PMC13073718; doi:10.3390/ijms27073079)
Supplement: Supplementary file 1 [file ijms-27-03079-s001.zip › ijms-4191543-supplementary figures.pdf]

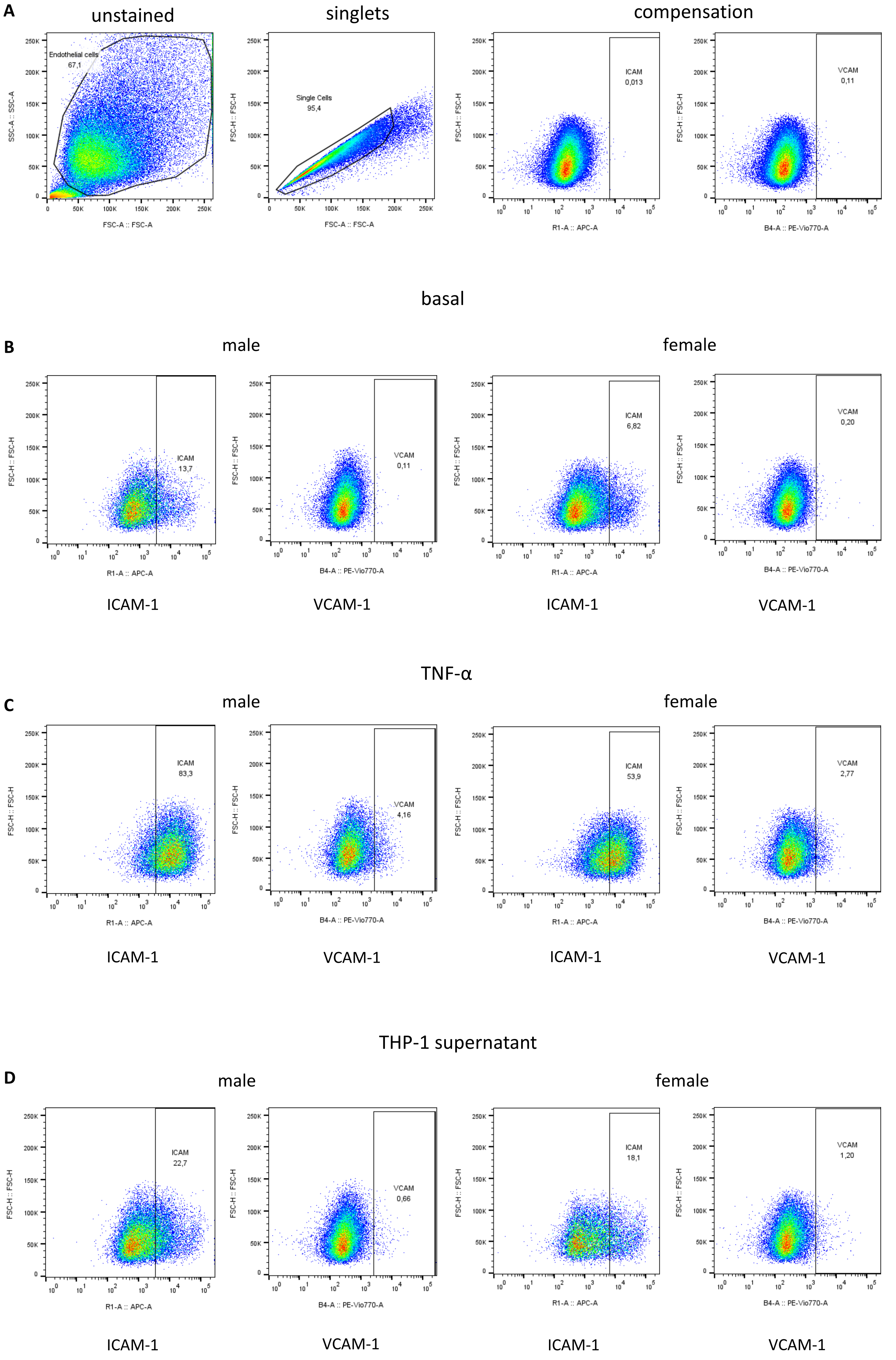

Figure S1: Flow cytometric analysis of the ICAM-1 and VCAM-1 expression in pro-inflammatory HUVEC. (A) Gating and compensation strategy. Representative plots of ICAM-1 and VCAM-1 expression in male and female (B) untreated HUVEC, (C) HUVEC treated with TNF-α (10 ng/ml), or (D) HUVEC stimulated with supernatant from pro-inflammatory THP-1 cells (1:1 dilution in cell culture medium) (n = 10).

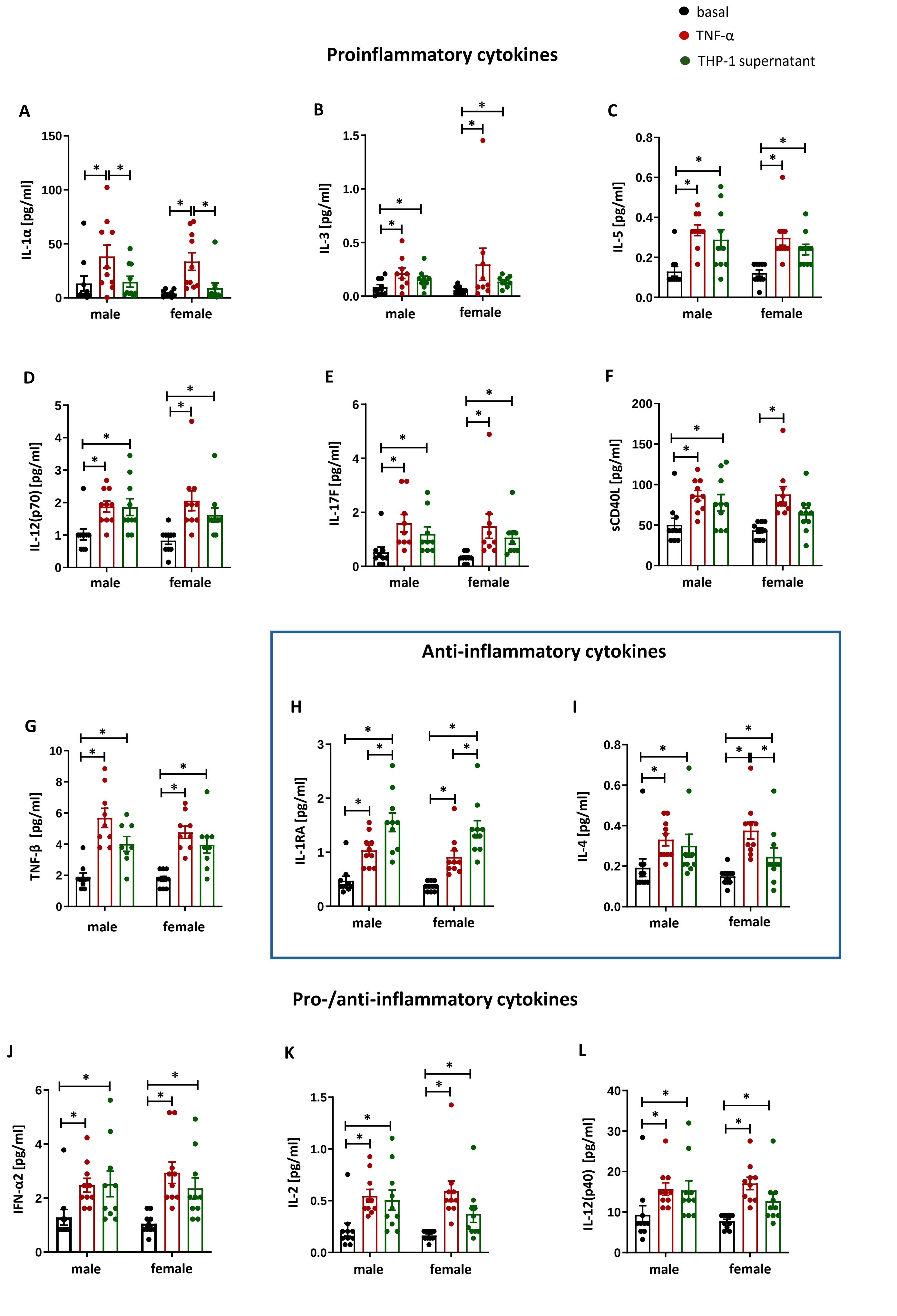

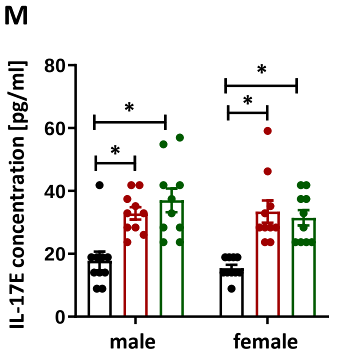

Figure S2: Secretion of pro- and anti-inflammatory cytokines is increased in activated male and female HUVEC. Male and female HUVEC were treated with TNF-α (10 ng/ml) or supernatant from pro-inflammatory THP-1 cells (1:1 dilution in cell culture medium) for 24 h. MAGPIX® analyses of (A) IL-1α, (B) IL-3, (C) IL-5, (D) IL-12(p70), (E) IL-17F, (F) sCD40L, (G) TNF-β, (H) IL-1RA, (I) IL-4, (J) IFN-α2, (K) IL-2, (L) IL-12(p40) and (M) IL-17E in the supernatants. Data are presented as bar charts with individual data points and means ± SEM (n = 10). All data were normalized to the mean of the male basal control. Statistical analysis was performed using the paired Wilcoxon test. *$p < 0.05$. basal: control; TNF-α: TNF-α treatment; THP-1 supernatant: THP-1 supernatant treatment.

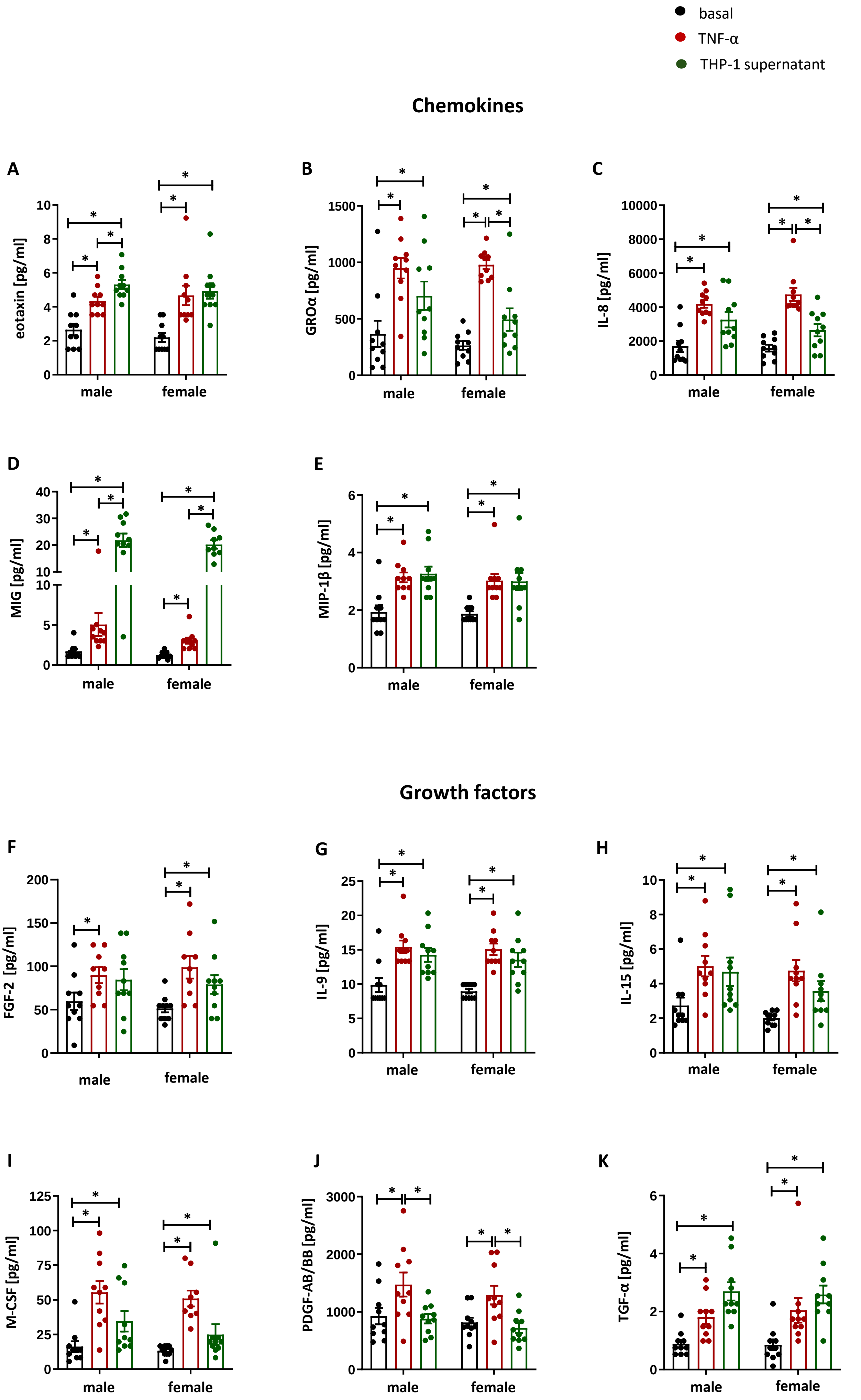

Figure S3: Higher secretion of chemokines and growth factors in activated male and female HUVEC. Male and female HUVEC were treated with TNF-α (10 ng/ml) or supernatant from pro-inflammatory THP-1 cells (1:1 dilution in cell culture medium) for 24 h. MAGPIX® analyses of (A) eotaxin, (B) GROα, (C) IL-8, (D) MIG, (E) MIP-1β, (F) FGF-2, (G) IL-9, (H) IL-15, (I) M-CSF, (J) PDGF-AB/BB, and (K) TGF-α in the supernatants. Data are presented as bar charts with individual data dots and means ± SEM (n = 10). All data were normalized to the mean of the male basal control. Statistical analysis was performed using the paired Wilcoxon test. *$p < 0.05$. basal: control; TNF-α: TNF-α treatment; THP-1 supernatant: THP-1 supernatant treatment.
