# Supplementary material for: Pro-Inflammatory Activation Promotes Atherogenic Endothelial Phenotype in Male and Female Human Umbilical Endothelial Vein Cells (HUVECs)
Source: Int J Mol Sci. 2026 Mar 27;27(7):3079. doi: 10.3390/ijms27073079 (PMC13073718; doi:10.3390/ijms27073079)
Supplement: Supplementary file 1 [file ijms-27-03079-s001.zip › ijms-4191543-supplementary table.pdf]

Table S1. Overview of the Real-Time PCR primer.

SYBR™ Green primer

RNA-primer	Primer Sequence<br>(f: forward, r: reverse)	Manufacturer
HPRT	f: 5‘- CTT TGC TGA CCT GCT GGA TT-3‘<br>r: 5‘- TAT GTC CCC TGT TGA CTG GT-3‘	Eurofins Genomics (Ebersberg, Germany)
RPL0	f: 5‘- ACG GGT ACA AAC GAG TCC TG-3‘<br>r: 5‘- AGC CAC AAA GGC AGA TGG AT-3‘	Eurofins Genomics (Ebersberg, Germany)
ICAM-1	f: 5‘- CCT TCC TCA CCG TGT ACT GG-3‘<br>r: 5‘-AGC GTA GGG TAA GGT TCT TGC-3‘	Thermo Fisher Scientific (Waltham, USA)
VCAM-1	f: 5’-TGC ACA GTG ACT TGT GGA CAT-3’<br>r: 5’-CCA CTC ATC TCG ATT TCT GGA -3’	Thermo Fisher Scientific (Waltham, USA)

TaqMan primer

RNA-primer	Assay ID	Manufacturer
α1-Actinin	Hs00998100_m1	Applied Biosystems (Foster City, USA)
FAK (PTK2)	Hs01056457_m1	Applied Biosystems (Foster City, USA)
HPRT	Hs02800695_m1	Applied Biosystems (Foster City, USA)
Paxillin	Hs01104424_m1	Applied Biosystems (Foster City, USA)
RPL0	Hs00420895_gH	Applied Biosystems (Foster City, USA)
Talin-I	Hs00196775_m1	Applied Biosystems (Foster City, USA)
Tensin-II	Hs00539247_m1	Applied Biosystems (Foster City, USA)
Vinculin	Hs00419715_m1	Applied Biosystems (Foster City, USA)
